# A Case Report of a Rare Cervical Synovial Cyst at the C7-T1 Level

**DOI:** 10.7759/cureus.83874

**Published:** 2025-05-11

**Authors:** Angel Parushev, Bogomil Iliev, Deyan Dzhenkov, Nadezhda Stefanova, Yanko G Yankov, Mustafa Ali, Yavor Enchev

**Affiliations:** 1 Department of Neurosurgery and ENT, Medical University "Prof. Dr. Paraskev Stoyanov", Varna, BGR; 2 Clinic of Neurosurgery, University Hospital "St. Marina", Varna, BGR; 3 Department of General and Clinical Pathology, Forensic Medicine and Deontology, Medical University "Prof. Dr. Paraskev Stoyanov", Varna, BGR; 4 Clinic of General and Clinical Pathology, University Hospital "St. Marina", Varna, BGR; 5 Department of General and Operative Surgery, Medical University "Prof. Dr. Paraskev Stoyanov", Varna, BGR; 6 Clinic of Maxillofacial Surgery, University Hospital "St. Marina", Varna, BGR

**Keywords:** cervical spine disorders, cervical synovial cyst, hemilaminectomy, laminectomy, mri, numbness in the limbs

## Abstract

Synovial cysts are rare, non-neoplastic lesions, with occasional cervical spine involvement. These cysts may compress neural structures, causing radicular pain, myelopathy, or neurological deficits. Surgical excision is the standard treatment for symptomatic cases. This case report presents a 52-year-old man with a three-month history of neck pain, stiffness, and progressive left upper limb weakness and numbness. Magnetic resonance imaging (MRI) revealed a left-sided flaval ligament cyst at C7, causing spinal cord compression and myelopathy. Surgical excision was successfully performed via a posterior midline approach. Histopathological findings confirmed a hemorrhagic synovial cyst. Postoperatively, the patient experienced full symptom resolution. Although rare, synovial cysts should be considered in the differential diagnosis of extradural spinal lesions, as their presence carries significant implications for surgical management.

## Introduction

Synovial cysts are cystic lesions characterized by a synovial lining and are in continuity with the facet joint [1]. These lesions are non-neoplastic and arise from herniation of the synovium through the joint capsule or tendon sheath into adjacent tissues or from the expansion of a pre-existing bursa [2].

In the spine, synovial cysts are often associated with conditions such as osteoarthritis, spinal instability (including spondylolisthesis), and trauma [2]. The first description of this entity dates back to 1880, marking the initial recognition of its clinical and pathological characteristics [3]. Synovial cysts most commonly occur in the lumbar spine, with thoracic and cervical presentations being significantly less frequent [4,5]. Cervical spinal synovial cysts are particularly rare, representing only 2.6% of all spinal synovial cysts [6]. Although uncommon, these cysts can cause spinal cord or nerve root compression, leading to symptoms consistent with mass effect. As they enlarge, synovial cysts may become symptomatic by exerting pressure on neural elements within the spinal canal [7]. For extradural synovial cysts, the current standard of care involves surgical decompression through laminectomy and cyst excision [8].

This case report presents a case of a cervical synovial cyst recently treated in the Clinic of Neurosurgery at the University Hospital "St. Marina", Varna, Bulgaria. Informed consent was obtained from the patient. Additionally, we conducted a review of the relevant literature for this rare entity, focusing on contemporary management strategies and decision-making approaches for this rare condition.

## Case presentation

In August 2024, a 52-year-old man was admitted to the Clinic of Neurosurgery at the University Hospital "St. Marina", Varna, Bulgaria. The patient presented with a three-month history of neck pain and stiffness, which had worsened over the past month, accompanied by left upper limb weakness, paresthesia, and pain. He did not report any concomitant disease or any medication to be taken regularly.

Physical examination demonstrated a reduced range of motion in the cervical spine due to pain and stiffness. Clinical findings were consistent with right C8 radiculopathy, evidenced by sensory deficits in the C8 dermatome and weakness in grip strength and finger flexion on the affected side. The biceps reflex was preserved, and tone and reflexes in the lower limbs were symmetrical.

**Figure 1:**
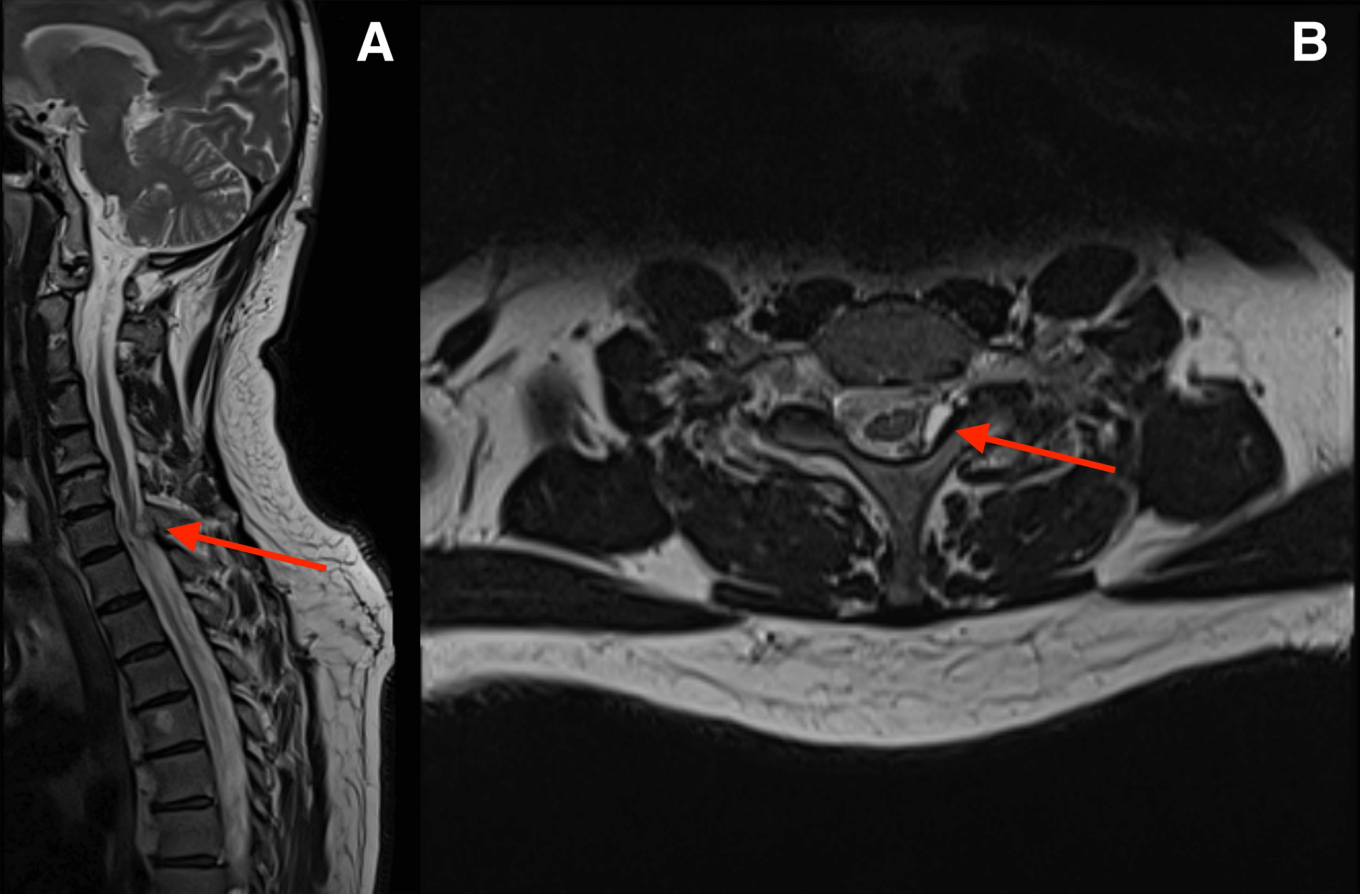
Cervical spine MRI A: Sagittal MRI showing a cervical synovial cyst at the C7-T1 level (red arrow). B: Axial MRI demonstrating an intraspinal, extramedullary cyst located left parasagittally, causing spinal cord compression and associated myelopathy (red arrow). MRI: magnetic resonance imaging

A cervical magnetic resonance imaging (MRI) scan demonstrated a cyst-like lesion (dimensions: 7.3×9×5.3 mm) of the flaval ligament at the level of C7 vertebrae on the left side, causing compression and myelopathy of the spinal cord in the zone of indentation, with noted cervical lordosis, cervicoarthrosis, and mild multisegmental degenerative discogenic pathology with specific characteristics and localization (Figure 1).

**Figure 2:**
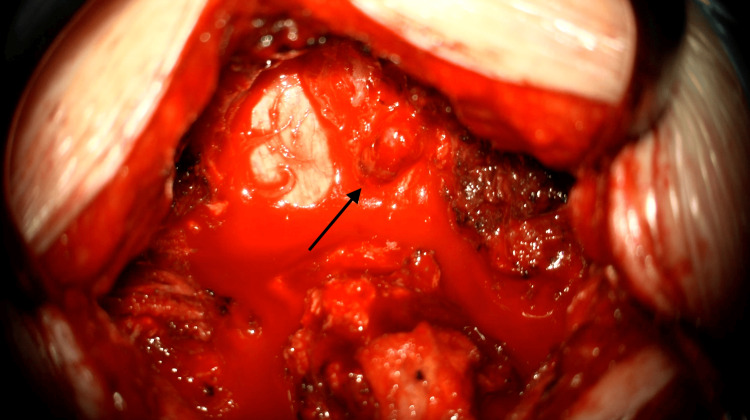
Intraoperative image visualizing the cyst following left hemilaminectomy at the C7 level (black arrow)

Considering these findings, the patient provided written informed consent and underwent surgical excision of the cyst under general anesthesia. A left C7 hemilaminectomy was performed via a posterior midline approach. While most reported cases describe a full laminectomy, we opted for a hemilaminectomy, which provided adequate exposure to visualize and access the lesion while preserving spinal stability. The cyst was visualized under the microscope and found to originate from the left C7-T1 facet joint. Precise excision of the cyst was successfully accomplished using a microsurgical technique, ensuring meticulous removal of the cyst wall while preserving surrounding structures (Figure 2). We decided against using fusion in this case due to the absence of cervical instability on imaging and patient-specific factors, such as age and lack of comorbidities.

**Figure 3:**
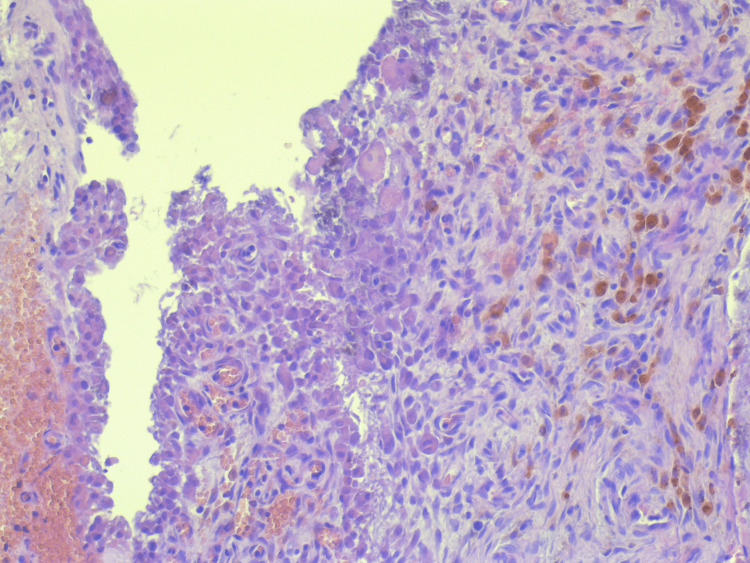
Histological sections show a synovial cyst lined by a cuboidal epithelium-like fibrous wall, with focal hemosiderophages and areas of dystrophic calcification (H&E, ×10) H&E: hematoxylin and eosin

The histological analysis of the excised mass revealed characteristic features consistent with a synovial cyst. The examination identified fragmented sections of the cystic wall, lined with synovial cells, alongside areas of fibrous connective and cartilaginous tissue interspersed with small foci of calcifications. Extensive zones of hemorrhage were observed, surrounded by perifocally located pigmented macrophages, identified as siderophages. These histopathological findings confirmed the diagnosis of a hemorrhagic synovial cyst (Figure 3).

The postoperative period was uneventful, with immediate resolution of the patient's radicular pain following the procedure. The patient demonstrated good neurological recovery, with improvement in grip strength and sensory function in the affected C8 distribution. Early mobilization was encouraged, and the patient was started on a tailored physiotherapy program to optimize cervical spine mobility, strengthen upper limb musculature, and prevent post-surgical stiffness. Pain was well-controlled with analgesics, and no complications were observed during the hospital stay. The patient was discharged four days after surgery, with instructions to continue physiotherapy and attend regular follow-up appointments to monitor recovery and ensure long-term functional improvement. The patient was evaluated 10 days postoperatively for suture removal and assessment of wound healing, which demonstrated proper healing without complications. A subsequent follow-up at one month revealed complete resolution of neurological symptoms, confirming a successful surgical outcome.

## Discussion

Cervical synovial cysts are rare in adults and have not been documented in pediatric or adolescent populations [9]. Nevertheless, there is one case of a 16-year-old girl with a C6-C7 synovial cyst [10]. However, spinal synovial cysts most commonly present during the sixth decade of life [11]. To date, the natural history and pathogenesis of intraspinal synovial cysts remain poorly understood and require further elucidation [12].

Patients may be asymptomatic, and cysts can be discovered incidentally [13]. When cysts grow epidurally into the spinal canal, they can compress neural structures, leading to various clinical symptoms [2]. The clinical presentation of a cyst is influenced by its size, location, and the relationship to surrounding bony and neural structures. Most symptomatic patients report experiencing radicular pain and neurological deficits [4]. These cysts can bleed and have a hemorrhage inside them, leading to the sudden onset of symptoms or their increased volume, identical to our case presentation [14].

Most reported cases of cervical synovial cysts have been located at the C7-T1 level [15]. A recent comprehensive study conducted in 2023 analyzed 96 cases of cervical synovial cysts reported in the literature, categorizing them according to their cervical spine level as follows: C2-C3 (four cases), C3-C4 (11 cases), C4-C5 (12 cases), C5-C6 (seven cases), C6-C7 (seven cases), and C7-T1 (55 cases) [16]. MRI is the imaging modality of choice for diagnosing spinal synovial cysts [17]. On MRI, these cysts appear as well-defined, extradural, intraspinal lesions typically situated adjacent to the facet joint [1].

In symptomatic patients with refractory pain and neurological symptoms, surgical management is advised. According to a systematic review by Bydon et al., spinal synovial cysts recurred in 1.8% of cases treated with decompression alone, while no recurrences were reported when fusion was added [18]. The primary surgical techniques employed included laminectomy and hemilaminectomy, with some cases incorporating fusion. However, the role of motion segment fusion in the management of symptomatic synovial cysts requires further investigation [19].

## Conclusions

Although rare, synovial cysts should be considered in the differential diagnosis of extradural spinal lesions, particularly in cases presenting with radiculopathy or neurological deficits. Early identification using appropriate imaging and management is crucial to prevent progressive neurological impairment and optimize patient outcomes. In symptomatic cases with neurological deficits, surgical intervention is recommended, with hemilaminectomy and laminectomy serving as the primary operative techniques.

## References

[1] Kwee RM, Kwee TC (2021). Imaging of facet joint diseases. Clin Imaging.

[2] Khan AM, Girardi F (2006). Spinal lumbar synovial cysts. Diagnosis and management challenge. Eur Spine J.

[3] Baker WM (1994). On the formation of synovial cysts in the leg in connection with disease of the knee-joint. 1877. Clin Orthop Relat Res.

[4] Themistoklis KM, Papasilekas TI, Boviatsis KA (2018). Spinal synovial cysts. A case series and current treatment options. J Clin Neurosci.

[5] Bydon M, Lin JA, de la Garza-Ramos R (2014). The role of spinal fusion in the treatment of cervical synovial cysts: a series of 17 cases and meta-analysis. J Neurosurg Spine.

[6] Yang DB, Harms J, Iyer RK, Arnold P (2023). Synovial cysts at the cervicothoracic junction: illustrative series of three cases. Surg Neurol Int.

[7] Mustroph ML, Cerecedo-Lopez CD, Groff M, Zaidi HA (2019). Bilateral synovial cysts as a rare cause of myelopathy in a 38-year-old woman. Cureus.

[8] Radhouane K, Dridi H, Mansouri N, Yedeas MD, Harbaoui A, Chkili R (2020). Hemorrhagic synovial cyst: an unexpected cause of acute cervical spinal cord compression. Case report. Int J Surg Case Rep.

[9] Found E, Bewyer D (2011). Cervical synovial cyst: case report. Iowa Orthop J.

[10] Overvliet G, van Scherpenzeel-de Vries MA, Wattjes MP, Vermeulen RJ (2015). Cervical synovial cyst in a 16-year-old girl. Pediatr Neurol.

[11] Kim DS, Yang JS, Cho YJ, Kang SH (2014). Acute myelopathy caused by a cervical synovial cyst. J Korean Neurosurg Soc.

[12] Gulensoy B (2022). Case report: a cervical synovial cyst. ODU Med J.

[13] Yarde WL, Arnold PM, Kepes JJ, O'Boynick PL, Wilkinson SB, Batnitzky S (1995). Synovial cysts of the lumbar spine: diagnosis, surgical management, and pathogenesis. Report of eight cases. Surg Neurol.

[14] Howling SJ, Kessel D (1997). Case report: acute radiculopathy due to a haemorrhagic lumbar synovial cyst. Clin Radiol.

[15] Lyons MK, Atkinson JL, Wharen RE, Deen HG, Zimmerman RS, Lemens SM (2000). Surgical evaluation and management of lumbar synovial cysts: the Mayo Clinic experience. J Neurosurg.

[16] Kido Y, Kamei N, Fujioka Y, Nakamae T, Adachi N, Sasaki M (2023). Microcervical foraminotomy for cervical juxtafacet cysts: case series and literature review. Int J Spine Surg.

[17] Dahuja A, Dahuja G, Kaur R (2015). Rare thoracolumbar facet synovial cyst presenting as paraparesis. Korean J Spine.

[18] Bydon A, Xu R, Parker SL, McGirt MJ, Bydon M, Gokaslan ZL, Witham TF (2010). Recurrent back and leg pain and cyst reformation after surgical resection of spinal synovial cysts: systematic review of reported postoperative outcomes. Spine J.

[19] Phan K, Mobbs RJ (2016). A rare case of cervical facet joint and synovial cyst at C5/C6. J Clin Neurosci.

